# Improving Access to Outpatient Antibiotic Therapy: Addition of Dalbavancin to a County Hospital Formulary for Complicated Staphylococcus aureus Infections

**DOI:** 10.7759/cureus.99450

**Published:** 2025-12-17

**Authors:** Saira Butt, Molly Tieman, Amy B Kressel, Bree Weaver, Michael Kays

**Affiliations:** 1 Internal Medicine/Infectious Diseases, Indiana University School of Medicine, Indianapolis, USA; 2 Pharmacology, Eskenazi Health, Indianapolis, USA; 3 Medicine/Infectious Diseases, Indiana University School of Medicine, Indianapolis, USA

**Keywords:** complicated staphylococcus aureus infections, cost savings, dalbavancin, electronic medical record, methicillin resistant staphylococcus aureus (mrsa)

## Abstract

Complicated *Staphylococcus aureus* infections often require prolonged intravenous (IV) antibiotic therapy, which poses challenges for patients, particularly those with unstable housing or substance use disorders, and leads to increased healthcare costs. Logistical barriers, including frequent lab monitoring and the need for nurse visits, add complexity to traditional outpatient antibiotic therapy. This study describes the integration of dalbavancin, a long-acting lipoglycopeptide antibiotic, into a county hospital's formulary as an outpatient therapy alternative for patients unsuitable for standard IV therapy. Dalbavancin has demonstrated clinical efficacy in treating infective endocarditis and osteomyelitis. One of our local hospitals’ internal data indicated that dalbavancin's cost per patient is significantly lower than standard care. This article discusses dalbavancin administration implementation via an infusion center post-discharge. The therapy plan, including dosing instructions and coordination mechanisms, was integrated into the electronic medical record system to streamline administration.

## Introduction

Patients with *Staphylococcus aureus* (SA) bacteremia (SAB) typically require intravenous (IV) antibiotics for a minimum of two to four weeks and additional time (six weeks) for infective endocarditis (IE) or osteomyelitis (OM) [1]. Patients generally receive IV therapy inpatient and then transfer to a facility or are discharged home with a long-term IV catheter for the duration of therapy. Patients deemed unsuitable for an IV catheter outpatient remain in the hospital or facility until the antibiotic course is completed, increasing healthcare costs. Potential barriers to outpatient antibiotic therapy include people who inject drugs (PWID), frequent lab monitoring, and nurse visits (at least weekly) [2]. Although POET and OVIVA trials have shown non-inferiority of oral antibiotics for IE and OM, in practice, this is hard to accomplish in patients with certain vulnerable social situations, including PWID and unstable housing [3-5]. Long-acting injectable medicines for oral contraception, diabetes mellitus, and HIV offer patient choice and benefits for some individuals over the available oral medications [6]. Dalbavancin is a lipoglycopeptide antibiotic with a half-life of up to 346 hours, allowing for once-weekly dosing. It is indicated for acute bacterial skin and structure infections caused by designated Gram-positive organisms, including SA [7]. In a retrospective study, dalbavancin used for IE showed clinical cure in 92.6% of patients [8]. In another study of OM, clinical response was similar in the dalbavancin group at day 21 (94%), six months, and one year (96%) compared to the standard of care [9]. Among PWID, dalbavancin use led to significantly improved outcomes, including a higher clinical cure rate, lower readmission rate, and shorter hospital length of stay, which offset the cost of the drug [10].

## Materials and methods

We initiated discussions about adding dalbavancin to our county hospital formulary during our Antimicrobial Stewardship (AS) program’s committee meeting. We emphasized how dalbavancin could have reduced the length of stay for the small but non-zero number of patients who could not be treated successfully with our standard-of-care IV or oral antibiotics. We highlighted the utilization of expanding Medicare Advantage insurance plans that often provided comprehensive benefits beyond Original Medicare, including coverage for prescription drugs like antibiotics and access to a network of healthcare providers for outpatient services [11]. To learn from our university hospital's dalbavancin use, we hosted a pharmacist-led presentation to review their clinical data and cost analysis experience. Their unpublished internal review showed that the cost per patient on dalbavancin was $34,660 versus the standard care cost of $132,255 [12]. In their experience, prior authorization was not a problem because the insurers understood the cost-benefit. We dispelled a myth that a barrier to dalbavancin adoption at our hospital was reimbursement if it was received in the infusion center on the same day as hospital discharge. This barrier could have been mitigated if reimbursed as a separate encounter in an outpatient setting. In other words, a successful dalbavancin program required a reliable coordination process between the inpatient team, the Outpatient Parenteral Antimicrobial Therapy (OPAT) team, and the infusion center. Based on these discussions, our request to add dalbavancin to the formulary was approved by the Pharmacy and Therapeutics (P&T) committee. Next, we discussed indications for dalbavancin use at our hospital. Dalbavancin became an alternative for patients with complicated SA infection when four to six weeks of IV or alternative oral antibiotics were not feasible, as determined by the ID team. The AS committee recommended limiting dalbavancin's use to outpatient only and suggested creating a workflow of discharging patients directly to the outpatient infusion center for their dose of dalbavancin. The dalbavancin therapy plan was created in the electronic medical record (EMR).

Dalbavancin therapy plans and education, including a frequently asked questions document (Table 1), were shared with the appropriate teams. The ID inpatient team coordinated with the inpatient social worker and the outpatient Infusion Center to identify the exact discharge day. When ready to order, the ID team used the ID therapy plans in the EMR, selected dalbavancin 1500 mg on Day 1 and 1500 mg on Day 8, and chose 'accept'. Based on their knowledge of discharge plans, the ordering physician chose the start date and selected the relevant nursing access order based on the patient’s needs. The signed therapy plan was routed to the Infusion Center to finalize the chair space. Ultimately, additional coordination was needed to set a start date. The OPAT nurse manager helped coordinate the start date and scheduled the second dose for the following week. The therapy plan remained as a banner on the patient’s chart for the duration of the treatment course.

**Table 1:** Dalbavancin Pharm-A-Gram: Frequently Asked Questions

Frequently Asked Questions
Q: What is dalbavancin? Dalbavancin is a lipoglycopeptide antibiotic that can be used in the treatment of infections caused by Gram-positive microorganisms. Dalbavancin is FDA-approved for the treatment of skin and soft tissue infections and can additionally be used off-label for the management of endocarditis, osteomyelitis, and prosthetic joint infections. Dalbavancin binds to the D-alanyl-D-alanine terminus of the stem pentapeptide in nascent cell wall peptidoglycan, preventing cross-linking and interfering with cell wall synthesis. It has a long half-life (~346 hours), which allows for less-frequent dosing and makes it an appealing choice for patients requiring parenteral therapy.
Q. Who should dalbavancin be considered in? Dalbavancin is restricted to initiation by Infectious Diseases only and can only be used in the outpatient setting. Dalbavancin’s place in therapy is as an alternative to traditional IV therapy. It should be considered in patients who would be unsuitable for line placement, patients who cannot receive outpatient parenteral antimicrobial therapy (OPAT), or patients who inject IV drugs but cannot complete IV standard of care therapy. In addition, it could be considered for those who have failed oral therapy, patients in whom oral absorption of medications is limited, or there are other contraindications to oral antibiotics.
Q. What are the benefits of using dalbavancin? 1. Long half-life allows for improved adherence 2. Allows patients who cannot be treated with oral antibiotics or OPAT to discharge 3. Prevention of potential central line-related complications 4. Shorter hospital stays, which benefits both the patient and the hospital cost-wise 5. Allows the patient to return to their everyday life sooner, allowing them to resume work and get back to their families.
Q. How will dalbavancin be ordered? 1. Coordinate with Case Management, Inpatient Pharmacy, and the Infusion Center to determine discharge date. (a) The Infusion Center is only open Monday-Friday so no weekend discharges will be able to occur. (b) The Infusion Center will let the inpatient team know the availability of seats and help coordinate which date would be best for the therapy to begin. 2. This order will be placed by an Infectious Disease fellow or attending using the Epic ID Therapy Plan for Dalbavancin, which can be placed on either an inpatient or clinic encounter. Please see the ordering tip sheet for a step-by-step guide on how to order. 3. When ordering the therapy plan, please have the order begin on the day of discharge. 4. At the initial appointment, the Infusion Center will coordinate with the patient to schedule the next appointment. 5. If it is a holiday or weekend, the Infusion Center will be closed. Please coordinate so that discharge and subsequent doses do not fall on a holiday or a weekend day.
Q. What dose of dalbavancin should we use? There are several dosing strategies for dalbavancin that have been studied. 1. The most common and preferred regimen is 1500mg IV on day 1 and day 8 of treatment. 2. Alternative dosing regimens exist; please contact an ID pharmacist if you think an alternative dose is warranted.
Q. What else should I know about dalbavancin? 1. Infuse over 30 minutes via IV infusion. 2. Do not co-infuse dalbavancin with other medications or electrolytes. 3. If a common IV line is being used to administer other drugs and dalbavancin, ensure the line is flushed with 5% dextrose before and after each dalbavancin infusion. 4. The total time from reconstitution to dilution to administration should be <48 hours. 5. Dalbavancin is a more expensive antibiotic and should only be used by Infectious Diseases to ensure appropriate indication and practice financial stewardship.

## Results

Implementing dalbavancin into the EMR was conducted per the core competence of implementation science [13]. The process was characterized by multidisciplinary collaboration between pharmacists, infectious disease specialists, information technologists, and clinical informatics teams. Key steps included: (1) Formulary integration: Dalbavancin was added to the EMR medication database, with standardized dosing regimens reflecting current pharmacokinetic data [14,15]. (2) Order set development: Clinical decision support tools were created to guide appropriate use, including indications, dosing, and monitoring parameters, consistent with evidence-based practice. (3) Stakeholder engagement: Stakeholder meetings were held to ensure alignment with institutional AS goals and to address workflow integration, consistent with implementation science recommendations for multilevel engagement. (4) Training and support: Targeted training was provided to prescribers, pharmacists, and nursing staff, focusing on EMR navigation, order entry, and documentation requirements. Ongoing support was made available post-implementation. (5) Process evaluation: Implementation was assessed through an audit of EMR order entry accuracy, completeness of documentation, and adherence to institutional protocols. No patient-level data were available at this stage, so process measures were prioritized. (6) Barriers and facilitators: Key facilitators included strong leadership support, clear communication channels, and robust training resources. Barriers identified were initial unfamiliarity with dalbavancin dosing and workflow adjustments required for the initial dose, followed by the second weekly administration.

## Discussion

The successful implementation of dalbavancin into our county hospital's formulary represents a significant paradigm shift in the management of complicated SA infections. Our approach, which focused on a collaborative, data-driven strategy, provides a replicable model for other institutions facing similar challenges with long-term antibiotic therapy for vulnerable patient populations. Unlike the traditional model of prolonged inpatient stays or difficult-to-manage outpatient IV regimens, dalbavancin offers a pragmatic, long-acting alternative that directly addresses key barriers to care, such as unstable housing and a history of injection drug use. This initiative aligns with national AS goals by promoting the right antibiotic, for the right duration, in the right setting [16]. By reducing the need for prolonged inpatient care, we decrease healthcare costs, mitigate the risk of hospital-acquired infections, and improve patient quality of life. The data from our partner university hospital, showing a drastic cost reduction from $132,255 to $34,660 per patient, served as a powerful incentive and highlights the economic viability of this therapeutic strategy. This finding is particularly relevant for county hospitals, which often serve a disproportionate number of uninsured or underinsured patients and operate on tighter budgets. The successful adoption was not just about the drug itself but about the process. We focused on dispelling common misconceptions, such as reimbursement issues, and instead emphasized a seamless, coordinated workflow. By creating a standardized dalbavancin therapy plan in the EMR and fostering strong communication between inpatient ID teams, social workers, and the outpatient infusion center, we have created a system that prioritizes a safe and efficient transition of care. This is a crucial distinction from simply adding a new drug to the formulary; it is about creating a comprehensive patient-centered program. Looking ahead, our work lays the groundwork for further research.

This article outlines a successful implementation strategy but has several limitations. First, this is an implementation initiative and not a clinical trial; therefore, we do not present clinical outcomes or patient-specific data to prove efficacy or safety in our patient population. Future research should focus on a retrospective cohort study of patients treated with dalbavancin to assess clinical cure rates, recurrence, and readmission rates. Second, our program's success heavily depends on the financial reimbursement landscape, which is subject to change. While we succeeded with Medicare Advantage plans, coverage may vary with different insurers, and this needs to be continually monitored. Lastly, a new workflow requires sustained staff education and coordination to prevent errors and ensure consistent adoption. The long-term durability of the process outlined here has yet to be tested, and we could conduct a dedicated review of the workflow and its effectiveness annually.

## Conclusions

The successful introduction of dalbavancin to our county hospital formulary represents a pivotal advancement in managing complicated SA infections. This long-acting lipoglycopeptide antibiotic directly addresses critical logistical and social barriers, particularly for vulnerable patient populations like PWID or those with unstable housing, who may struggle with the demands of long-term outpatient IV antibiotic therapy. The implementation of a structured workflow in the EMR, along with strong inter-departmental coordination, provides a viable model for other institutions aiming to improve patient care and reduce healthcare costs. The significant cost savings demonstrated by our collaborating institution and the potential to reduce hospital length of stay further validate dalbavancin's utility as a strategic therapeutic option.
